# The application of 7H-indolo[1,2-a]quinolinium merocyanine as a new water sensor in organic solvents

**DOI:** 10.1007/s11696-017-0328-z

**Published:** 2017-10-28

**Authors:** Marta J. Sawicka, Elwira K. Wróblewska

**Affiliations:** Department of Physical Chemistry, Faculty of Chemical Technology and Engineering, West Pomeranian University of Technology Szczecin, Piastów Ave. 42, 71-065 Szczecin, Poland

**Keywords:** Water content, Water determination, Solvatochromic dye, Preferential solvation, UV–Vis spectroscopy

## Abstract

**Abstract:**

The 7H-indolo[1,2-a]quinolinium merocyanine was applied as a new water sensor in organic solvents such as ethanol, propane-1-ol, propane-2-ol, DMSO, and DMF. The spectral changes of the dye caused by the addition of increasing amount of water into an organic solvent were investigated. Based on the results, the calibration curves were found as a relation between the position of the absorption band of the dye and the water concentration ranging from about 0.05 to 11% (w/w). In case of ethanol, propane-1-ol and propane-2-ol the plots were linear, whereas in DMSO and DMF, better results were obtained with the use of a polynomial function. The method allowed to determine the water content in a fast and precise manner.

**Graphical Abstract:**

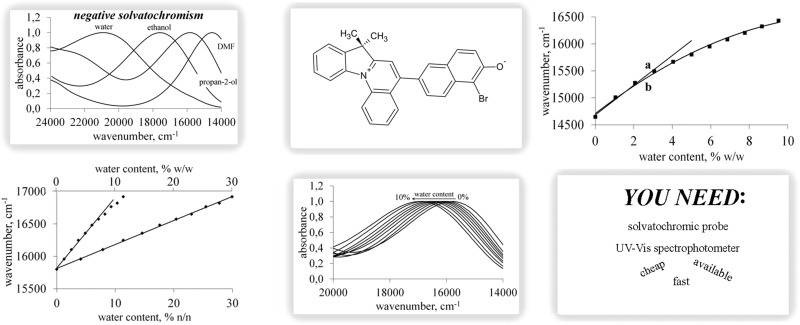

**Electronic supplementary material:**

The online version of this article (10.1007/s11696-017-0328-z) contains supplementary material, which is available to authorized users.

## Introduction

Determination of water content in organic solvents plays an important role in many laboratory and industry applications. Indeed, even small amount of water can change the rate and the direction of organic syntheses, the polarity of phases in chromatographic methods, the quality of fuel, etc.

The Karl Fischer titration and its modifications (Mitchell and Smith 1977; Scholz 1984; Liang 1990; Schöffski 2001; de Aquino et al. 2007) are the methods most often used for water determination in laboratory scale. Although its reliability and accuracy, Karl Fischer titration has many disadvantages (Bai and Yang 2007; Huang et al. 2015; Jung et al. 2016). It is time consuming and harmful to operator due to the contact with toxic reagents. It is also unsuitable in the presence of oxidants and significant amount of strong acids. Moreover, this method requires ex situ analysis, indicating its incapacity for real-time monitoring. Other methods which allow to determine the water in liquid and solid samples include gas chromatography (Iguchi et al. 1988; Nubbaum et al. 2000), liquid chromatography (Chen and Fritz 1989), infrared spectroscopy (Tran and Grishko 2004; Troshin et al. 2008; Bampi et al. 2013), refractometric method (Sanchez et al. 2010), microwave accelerated Dean–Stark method (Villet et al. 2009), electrochemical method (Gąsiorowska et al. 2009), as well as absorption and fluorescence spectroscopy (Bai and Yang 2007; Cha et al. 2011; Rahimi-Nasrabadi et al. 2015; Shen et al. 2016). Recently, the development of highly sensitive fluorescent and colorimetric molecular sensors seems to have great meaning. These sensors make possible in situ analysis of water content in organic solvents with high sensitivity and precision and low detection limits. Their applicability in water determination results from the fact that the addition of water into an organic solvent leads to the changes in intensities of their emission or absorption spectra and/or the spectral shifts of the sensors (Langhals 1990; Pinheiro et al. 2006; Cha et al. 2011; Rananaware et al. 2015a, b; Jung et al. 2016). Some examples of these compounds, as well as the mechanism of their action have been freshly described by Jung et al. (2016). Actually, fluorescent probes allow to obtain greater sensitivity than the colorimetric ones and are associated with lower detection limits. On the other hand, spectrofluorimeters are still much more expensive than the apparatus dedicated to UV–Vis absorption spectra measurements. For this reason, UV–Vis spectrophotometers seem to be much more popular either in analytical laboratories or these for organic synthesis. Therefore, the new water sensors are still wanted, which UV–Vis absorption spectra recorded in an organic solvent are sensitive to the presence of even small amount of water.

One group of compounds which can be used as the water sensors are merocyanines. These are systems in which an electron-donating group, *D*, is linked by a conjugated system, *R*, to an electron-accepting group, *A*. Their structure of a ground state can be described in terms of two mesomeric structures *D*–*R*–*A* ↔ *D* ^+^–*R*–*A* ^−^ with various dipole moments and solvent shell. The changes in electronic structure of the ground state of a merocyanine caused by the changes of solvent polarity, as well as the changes in the dipole moment during the excitation are responsible for the solvatochromism of these compounds, i.e., the phenomenon concerning the dependency of UV–Vis spectra on the polarity of the medium (Reichardt 2003). Among merocyanines, 7H-indolo[1,2-a]quinolinium dyes are worth mentioning (Soroka and Soroka 1980, 1989, 1991, 1997; Sawicka et al. 2006, 2010, 2012). These compounds have found many applications, for instance, in the determination of the ternary solvent mixture composition (Soroka and Soroka 2002; Sawicka and Soroka 2013), in investigations of the diffusion in polymers (Soroka et al. 2002; Wróblewska et al. 2005, 2016), as well as in quantitative analysis of ionic surfactants (Wróblewska et al. 2010; Wróblewska and Gąsiorowska 2011; Gąsiorowska and Wróblewska 2012a, b). In the whole group of 7H-indolo[1,2-a]quinolinium dyes, 5-(5-bromo-6-hydroxynaphthyl-2)-7,7-dimethyl-7H-indolo[1,2-a]quinolinium merocyanine (**1**) exhibits the best solvatochromic properties (Sawicka et al. 2012) which are comparable with these of Dimroth–Reichardt betaine 30 (Reichardt 2003).

Water is one of the most polar solvent (Reichardt 2003). Therefore, even small amount of it added to an organic solvent can distinctly influence the polarity of the system. As a consequence, in UV–Vis absorption spectra of a solvatochromic probe, a hypsochromic or bathochromic shift can be noticed. In most cases, the plot describing the relation between the position of the solvatochromic band maximum and the amount of water in an organic solvent is not linear in the whole range of water concentration (Langhals 1990; da Silva et al. 2002; Pinheiro et al. 2006; Cha et al. 2011). The deviation from the linearity can be explained in terms of the preferential solvation of the solute *S* (the solvatochromic probe) by one of the components of binary water–organic solvent mixtures. The preferential (or selective) solvation occurs when the solvent shell has a composition other than that in the bulk solution (Reichardt 2003). If the solute microenvironment is the same as the bulk composition, an ideal, i.e., a linear plot are obtained, since in microscopic and macroscopic terms, the polarity changes smoothly with the mixture composition. Moreover, when the solute microenvironment has more of one solvent than the other in comparison with the macroscopic ratio, the preferential solvation of the probe by one of the components occurs, and the deviation from linearity is observed (da Silva et al. 2002). Langhals (1990) tried to solve the problem with non-linear plots by the application of the two-parameter equation which was found to be a general description of the polarity of the binary mixtures as a function of their composition. The equation seemed to be quite complicated, and to found the water concentration in the organic sample, some empirical parameters were needed for each solvent. More recently, the changes in the UV–Vis absorption spectra of solvatochromic sensors caused by the changes in water content have been studied and the applicability of the probes was established in such a range of water concentration in which the linear relation was found between the absorbance or the absorption band position and the water content (Pinheiro et al. 2006; Cha et al. 2011).

In this paper 5-(5-bromo-6-hydroxynaphthyl-2)-7,7-dimethyl-7H-indolo[1,2-a]quinolinium merocyanine was presented as a new water sensor. The dye was successfully applied to the determination of water in common water miscible organic solvents, such as ethanol, propane-1-ol, propane-2-ol, DMSO, and DMF. Two kinds of calibration curve were proposed: the linear one which is typical for this kind of analysis and the parabolic one which in case of DMSO and DMF allowed to obtain very good results in wide range of water concentration.

## Experimental

### Materials

#### Chemicals

**Scheme 1:**
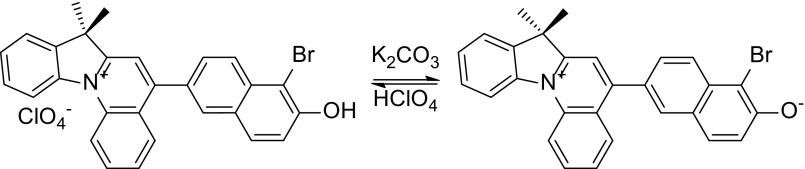
The formation of the merocyanine form of the dye

5-(5-Bromo-6-hydroxynaphthyl-2)-7,7-dimethyl-7H-indolo[1,2-a]quinolinium merocyanine **1** was generated in situ by alkalisation of the corresponding perchlorate solution (2 mL) with powdered anhydrous potassium carbonate (5 mg) (Scheme 1). 5-(5-Bromo-6-hydroxynaphthyl-2)-7,7-dimethyl-7H-indolo[1,2-a]quinolinium perchlorate was synthesized according to the method described in the literature (Sawicka et al. 2006, 2010).

K_2_CO_3_ was purchased from ChemLand (Stargard, Poland). Ethanol, propane-1-ol, propane-2-ol, DMSO, and DMF for spectroscopy were purchased from POCh (Gliwice, Poland) and were stored above molecular sieves. Distilled water was generated in glass Büchi apparatus and was used without additional purification.

#### Spectrophotometric measurements

UV–Vis absorption spectra were recorded with computer-controlled spectrophotometer Specord M40 (Carl Zeiss Jena, Germany) modified by Medson Electronics, Co Ltd. (Poznań, Poland) in the 12,000–27,000 cm^−1^ range at temperature 25 ± 0.1 °C. The resolution of the spectrophotometer was 20 cm^−1^. Gas-tight quartz cells with 1 cm path length were used.

Stock solutions of **1** (1.18 × 10^−2^ mol L^−1^) were prepared in an appropriate solvent or in a mixture of an appropriate solvent and DMSO (3:1). Next, the stock solution of dye (10 μL) was added to an appropriate solvent (2 mL) containing an incremental amount of water (20–200 μL). It was checked that the influence of the amount of DMSO used in the experiment on the shape and the position of absorption bands did not exceed the instrumental error.

Normalisation of the spectra was done in M48 set of programs (Medson Electronics, Co Ltd., Poznań, Poland). The applied procedure allowed to obtain the result of division of the stream by scalar of declared type.

## Results and discussion

### The changes in UV–Vis spectra

The 7H-indolo[1,2a]quinolinium merocyanines exhibit strong solvatochromism. It means that the alternations in polarity of the solvent lead to the changes in the intensity and the position of their absorption spectra. Most of 7H-indolo[1,2a]quinolinium derivatives show the negative solvatochromism in polar protic and aprotic solvents, i.e., a hypsochromic (blue) shift of their UV–Vis absorption band is observed when the polarity of the medium increases. The extent of this effect is not the same in case of all dyes belonging to this group. It was noticed that the merocyanines possessing naphthalene moiety are more sensitive to the changes in solvent polarity in comparison with the ones with phenyl ring (Sawicka et al. 2006). The best solvatochromic properties exhibits merocyanine **1**, the properties of which was studied in our earlier work (Sawicka et al. 2012). With a change of a solvent from trichloroethylene to water [from 150.8 to 264.2 kJ mol^−1^ in E_T_(30) polarity scale (Reichardt 2003)], the position of the absorption band of the dye changes from 12,400 to 20,640 cm^−1^. Since the dye exhibits really strong solvatochromism, one can expect that the addition of polar water into the dye solution in less polar solvent should lead to distinct spectral changes in the absorption spectra, which can be applied in the analysis of water content.

The studies of the influence of water on the UV–Vis spectra of **1** were conducted in five organic solvents differing in polarity. According to E_T_(30) scale, which is the most popular scale of solvent polarity, these solvents can be arranged from the most polar to the least polar as follows: ethanol (217.7 kJ mol^−1^), propane-1-ol (212.5 kJ mol^−1^), propane-2-ol (203.7 kJ mol^−1^), DMSO (188.6 kJ mol^−1^), and DMF (183.5 kJ mol^−1^) (Reichardt 2003).

**Fig. 1:**
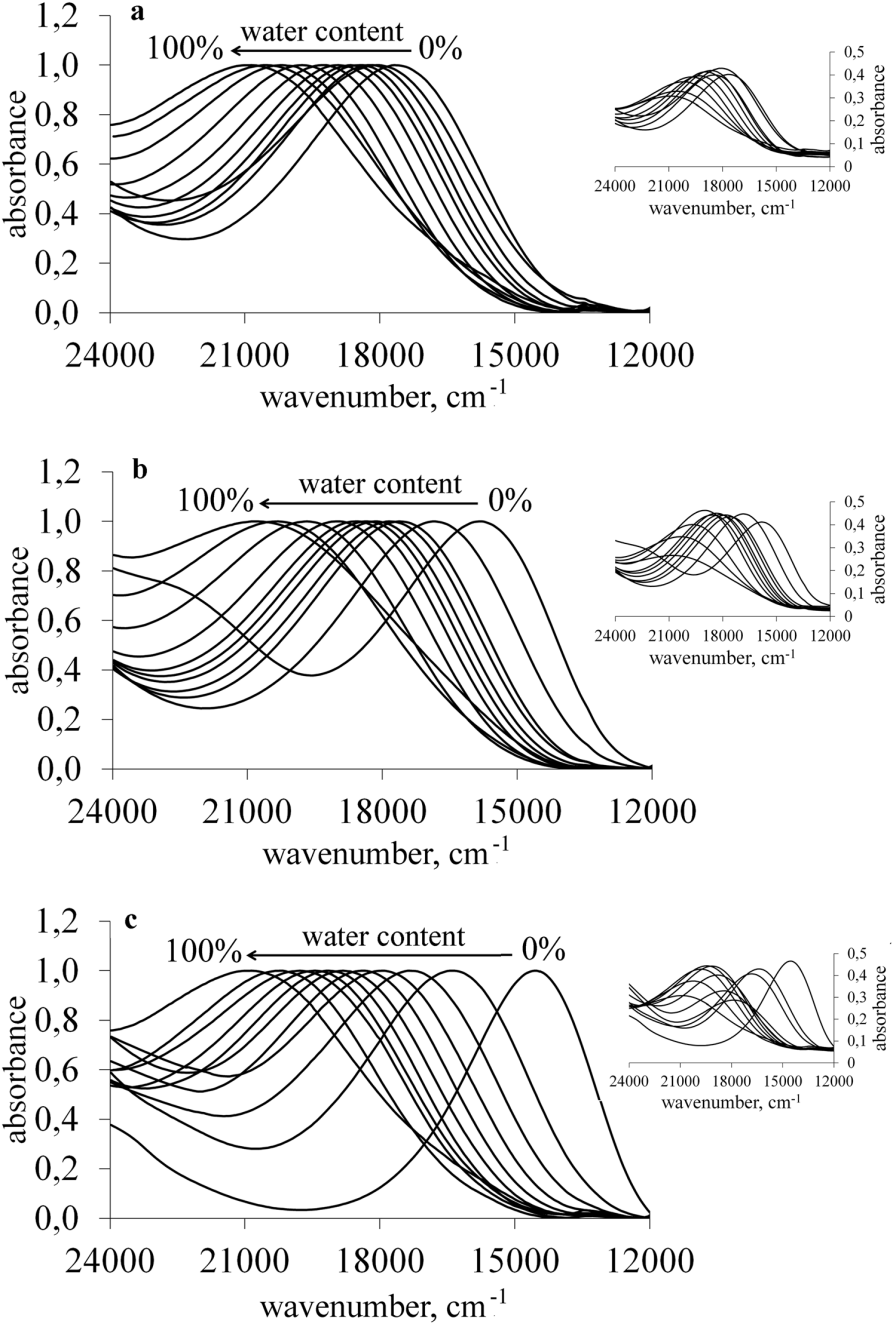
Original (inset) and normalised UV–Vis spectra of **1** recorded in ethanol (**a**), propane-2-ol (**b**), and DMF (**c**) at various water content

The addition of water and, as a consequence, the increase in the polarity of the medium caused hypsochromic shift of the merocyanine **1** absorption band recorded in all studied solvents. It was connected with the negative solvatochromism of the probe which resulted from the decrease of its dipole moment upon excitation. As a consequence, the ground state of the dye was better stabilised in comparison with the excited state with increasing medium polarity (Sawicka et al. 2006). Thus, due to the interaction with more polar solvent, the energy of the solute ground state was lowered greater than that of the excited state. As a result, the excitation energy rose and the blue shift was observed. The spectral changes were more pronounced in case of less polar solvents than in more polar ones. The sample spectra are shown in Fig. 1. The original spectra were normalised to show what was the range of the changes in the position of the absorption band.

**Fig. 2:**
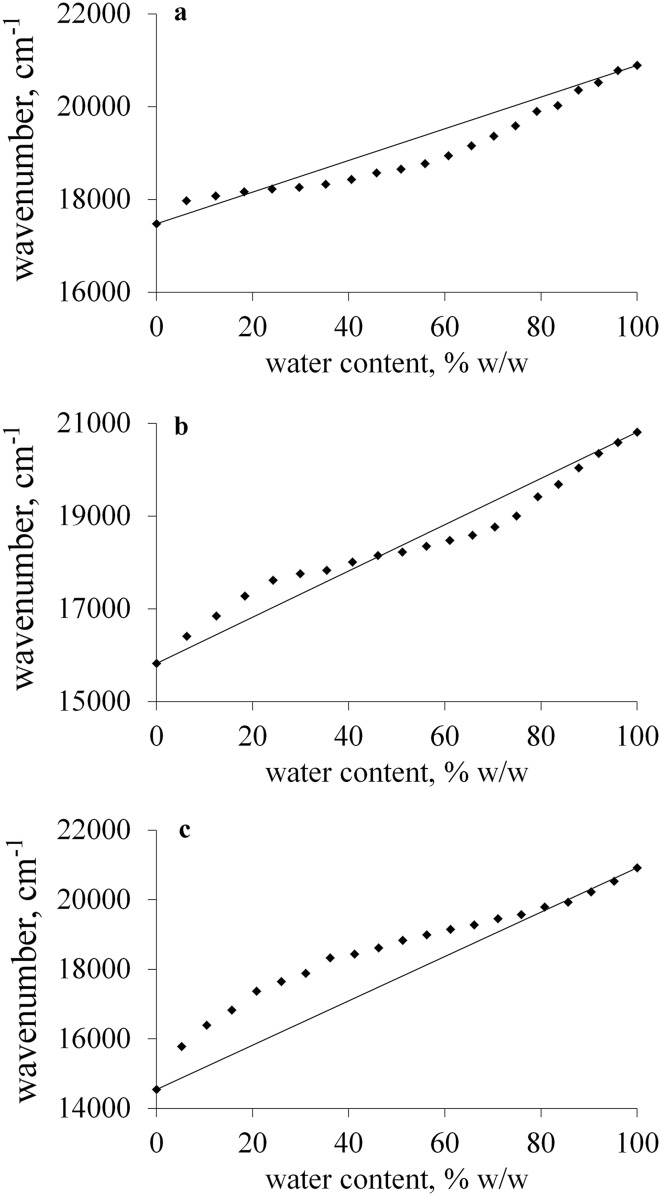
Influence of water on the position of the absorption band of **1** recorded in binary mixtures of water with ethanol (**a**), propane-2-ol (**b**), and DMF (**c**). Rhombuses indicate experimental points and the solid line represents the ideal linear behavior

The changes in the position of the absorption band of **1** as a function of water content are presented in Fig. 2. In all studied solvents, non-linear plots were obtained. The distribution of the experimental points can be clarified taking into account solute–solvent and solvent–solvent interactions that take place in binary water–organic solvent mixtures. Indeed, these two types of interactions influence the composition of the solvation shell which in some cases can differ from the bulk composition.

The shape of obtained plots implies that in ethanol, propane-1-ol and propane-2-ol the preferential solvation of the probe is observed by water at low water concentration and by alcohol at higher water content, whereas in case of DMF and DMSO in almost whole range of water content, the preferential solvation of the probe by water can be noticed. The extent of the preferential solvation of **1** by water increases in the order ethanol < propane-1-ol < propane-2-ol < DMSO < DMF. This is the same direction of the decrease of the polarity of the solvents as well as their ability of being hydrogen bond donor. If the polarities of water and an organic solvent are quite similar, the experimental points are located close to the ideal, i.e., linear behavior. When the difference between the polarities of water and an organic solvent is more evident, the preferential solvation of the probe by water is especially pronounced and the deviation from the linearity is distinctly observed. Based on the results, one can admit that the solvatochromic merocyanine has a greater tendency to interact with polar solvent than less polar one. On the other hand, it should be taken into account that the solvent–solvent interactions play an important role as well. In diluted water solutions, the molecules of organic co-solvent compete with the dye of free water molecules. In water–alcohol mixtures, the molecules of an alcohol are able to make hydrogen-bonded complexes with water, which were responsible for the solvation of the dye molecules. As a result, in such a system, the preferential solvation of the probe by water is observed in minor extent in comparison with water–DMSO and water–DMF mixtures. In binary mixtures of water and the aprotic and much less polar DMSO or DMF, the water prefers to interact with the molecule of the dye rather than with that of organic co-solvent and the preferential solvation is especially pronounced. On the other hand, at higher water concentration, the water molecules prefer to interact with itself and they tend to create strong hydrogen-bonded nets. As a result, the preferential solvation of the probe by an organic co-solvent is observed, but only when the solvent is a good hydrogen bond donor. Therefore, an alcohol is able to interact specifically with the dye molecule, whereas DMF or DMSO possess too weak affinity to the dye. This is the reason why in binary mixtures of water with DMF and DMSO, the preferential solvation by less polar co-solvent cannot be noticed.

Based on the results, an attempt was made to apply the solvatochromic merocyanine **1** in the determination of water in the studied solvents at the water content not exceeding 10% (v/v).

### Linear calibration plot

UV–Vis absorption spectra of **1** were measured in organic solvents in the presence of different amount of water ranging from 0 to 10% (v/v) and the positions of the absorption band were determined. The obtained values were plotted versus the water content in an organic solvent expressed as mass percent (%, w/w) which seems to be more adequate unit than volume percent in the analysis of mixtures of water with an organic solvent due to the volume contraction (the total volume of the solution should not be calculated as a sum of the volumes of pure components). The results of fitting the linear function to the experimental points are summarised in Table 1. The limit of detection (LOD) found for each solvent was presented in Table 1 as well. LOD is defined as the lowest concentration at which an analyte can be sensed over the noise with a high degree of certainty. It was calculated from three times of standard deviation of the blank. The found LOD values were comparable with those obtained with the use of other molecular sensors.

**Table 1:** Characteristic of the linear calibration plot $\bar{\nu }$ = *f*(%_water_, w/w)

	Ethanol	Propane-1-ol	Propane-2-ol	DMSO	DMF
Linear range/%,w/w	0.0–11.25	0.0–11.06	0.0–7.13	0.0–4.35	0.0–3.06
LOD/%,w/w	0.22 (0.001–1.3%)^a^	0.14 (0.23–0.51%)^b^	0.08 (0.001–0.054%)^c^	0.05 (0.004–1.1%)^d^	0.04 (0.002–0.16%)^e^
*a*	29.31	50.58	108.90	201.47	274.85
*R*	0.998	0.998	0.998	0.997	0.994

*LOD* the limit of detection, *a* the parameter in the equation *y* = *ax* + *b*

^a^Langhals (1990), Li and Pacey (1997), Dantan et al. (2000), Zhou et al. (2011) and Jung et al. (2016)

^b^Langhals (1990) and Ohira et al. (2012)

^c^Li and Pacey (1997) and Dantan et al. (2000)

^d^Langhals (1990) and Cha et al. (2011)

^e^Langhals (1990), Dantan et al. (2000), Deng et al. (2012) and Cigán et al. (2017)

It can be concluded from the data that the sensitivity of the method (the slope *a* of the linear plots), and as a consequence the LOD values, as well as the range of water concentration in which the linear relation was found differ in case of various solvents.

**Fig. 3:**
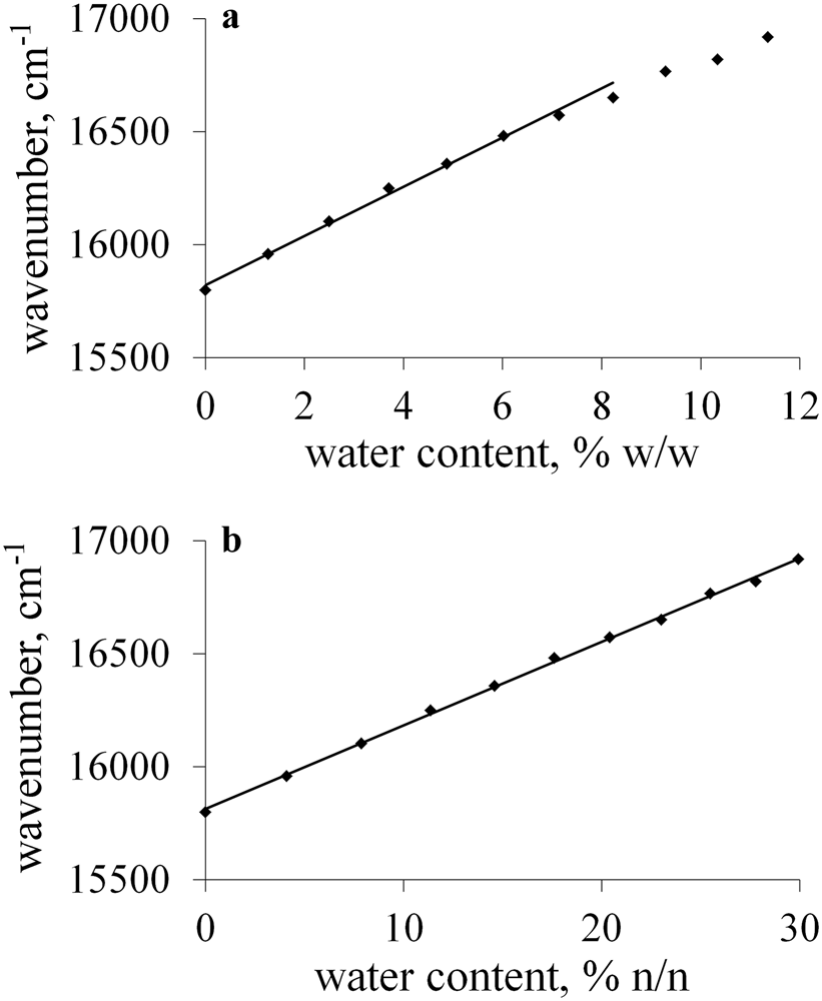
Calibration plots of the position of the absorption band of **1** in propane-2-ol versus water content expressed as mass (**a**) and mole (**b**) percent

The difference in the method sensitivity can be explained in terms of the difference in the polarity between two components of the binary mixture, since it influences the spectral shift of the probe recorded by going from an organic solvent to water. For example, the change of the solvent from ethanol to water leads to the hypsochromic shift of the absorption band of merocyanine **1** equals to about 3000 cm^−1^, whereas in case of DMF, the hypsochromic shift of more than 6000 cm^−1^ can be noticed. Therefore, the spectral shift is much more pronounced in case of less polar DMF than in case of polar ethanol. Moreover, the preferential solvation of the probe by more polar water seems to have great meaning as well. In diluted water solutions, due to the interactions between water and the sensor in nonpolar medium, the position of the UV–Vis absorption band of the dye approaches the value close to that recorded in pure water much faster than it is in polar solvent, in which the preferential solvation of **1** by water is less evident. Therefore, the best results were obtained for DMF, whereas the determination of water in ethanol was the least sensitive. On the other hand, the range of water content which can be determined using the method decreased in the opposite direction. In ethanol and propane-1-ol the plots were linear in the whole studied range of water content. However, as the water concentration exceeds 7, 4, and 3% for propane-2-ol, DMSO, and DMF, respectively, the plots demonstrated the deviation from linearity (Fig. 3a) which can be explained in terms of preferential solation of **1** by water.

Taking into account that the reason of observed spectral changes is connected with intermolecular solute–solvent and solvent–solvent interactions, the plots were determined describing the changes in the position of the absorption band of **1** in water–organic solvent mixtures as a function of water content expressed as mole percent (%, (n/n)). The effect of the unit conversion is presented in Fig. 3. The linear calibration curves were obtained in the whole studied range of water concentration for ethanol, propane-1-ol, and propane-2-ol. The ranges of water content in which the linear dependence was found in case of DMSO and DMF were much wider than that obtained for mass%.

The results of fitting the linear function to the experimental points are presented in Table 2. To make possible the comparison between the data obtained with the application of mass and mole percent of water, the values of mole% corresponding to the range of water content in which linear plots were found and to the detection limit were converted into the values of mass% according to the equation:

1 $${\text{mass}}\%_{\text{water}} = \frac{{1800 \cdot {\text{mole}}\%_{\text{water}} }}{{18 \cdot {\text{mole}}\%_{\text{water}} + (100 - {\text{mole}}\%_{\text{water}} ) \cdot M_{\text{org}} }}$$

where *M*_org_ is the molecular mass of organic solvent.

**Table 2:** Characteristic of the linear calibration plot $\bar{\nu }$ = *f*(%_water_, n/n)

	Ethanol	Propane-1-ol	Propane-2-ol	DMSO	DMF
Linear range/%,w/w	0.0–11.25	0.0–11.06	0.0–11.35	0.0–7.56	0.0–4.04
LOD/%,w/w	0.23	0.11	0.07	0.05	0.04
*a*	13.75	19.15	37.42	48.83	69.59
*R*	0.998	0.998	0.998	0.997	0.995

*LOD* the limit of detection, *a* the parameter in the equation *y* = *ax* + *b*

In the calculations, the molecular mass of water was accepted as 18.00 g mol^−1^ and the water density as 1.00 g cm^−3^.

### Parabolic calibration plot

The preferential solvation of the solvatochromic merocyanine **1** by water was the reason why the plots of the position of its absorption band versus water content obtained in the studied range of water concentration in DMSO and DMF demonstrated the deviation from linearity. The analysis of the obtained results indicated that the experimental points in these two solvents did not follow the linear function (*y* = *ax* + *b*), but the polynomial one (*y* = *ax* ^*2*^ + *bx* + *c*) the plot of which is parabola. The parabolic plot is not commonly used as a calibration curve which should be monotonic and give only one solution corresponding to the unknown concentration of an analyte. Indeed, these circumstances are typical for linear function, whereas a polynomial plot is not monotonic and, as a consequence, usually gives two solutions, which can be calculated as follows:

2 $$x_{1} = \frac{ - b + \sqrt \Delta }{2a}$$

and

3 $$x_{2} = \frac{ - b - \sqrt \Delta }{2a}$$

where Δ equals to *b* ^2^ − 4*ac.*

**Fig. 4:**
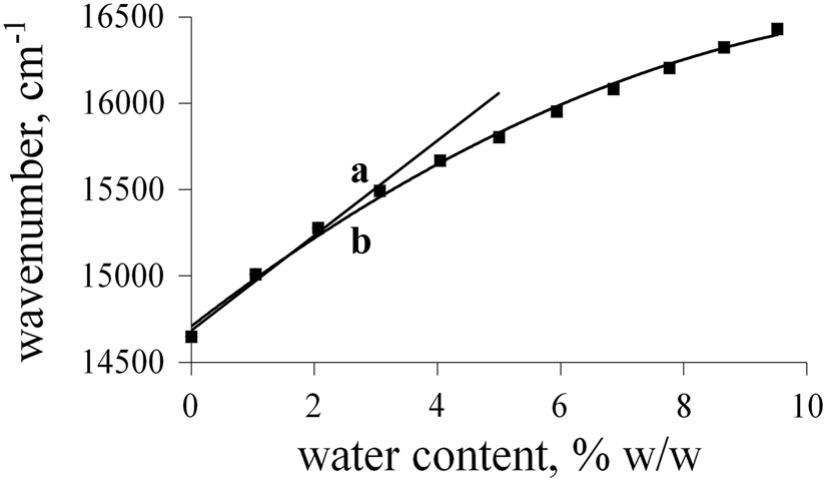
Linear (**a**) and polynomial (**b**) calibration plots describing the position of the absorption band of **1** in DMF versus water content

Despite these facts, the polynomial plot was successfully applied as a calibration curve of water content in DMSO and DMF (Fig. 4).

The fitting of the polynomial function to the position of the absorption band maximum of **1** measured in these two solvents allowed to obtain calibration curve in the whole studied range of water content with high value of correlation coefficient (Table 3). The plots were found as a function of water content expressed as both mass percent and mole percent with the correlation coefficients a little bit higher in the latter case.

**Table 3:** Characteristic of the polynomial calibration plot $\bar{\nu }$ = *f*(%_water_, w/w)

	DMSO	DMF
Water content/%,w/w	0.0–8.33	0.0–9.52
LOD/%,w/w	0.05	0.04
*R*	0.998	0.998

In the studied range of water content in DMSO and DMF, the experimental data were arranged in one arm of the parabola, and therefore, the polynomial function was monotonic and gave only one meaningful solution which was available from Eq. 3 (as *x*_1_). The second solution (*x*_2_) was out of the studied range of water concentration, i.e., was higher than 10% (w/w). Moreover, in some cases, *x*_2_ was bigger than 100% which was inconsistent with the definition of mass or mole percent (Table 4).

**Table 4:** The comparison of two possible solutions of the parabolic calibration plot of water content in DMF in the concentration range 0–9.52%, w/w (0–29.91%, n/n)

*x*_real_/%, w/w; n/n	*x*_1found_/%, w/w^a^	*x*_2found_/%, w/w^a^	*x*_1found_/%, n/n^b^	*x*_2found_/%, n/n^b^
1.04; 4.09	1.03	25.09	4.09	119.43
3.06; 11.35	3.11	23.00	11.40	112.12
5.00; 17.58	4.93	21.19	17.38	106.14
6.86; 23.00	6.90	19.22	23.25	100.27
8.65; 27.75	8.65	17.47	27.63	95.89

^a^Calculated based on the plot $\bar{\nu }$ = *f*(%_water_, w/w)

^b^Calculated based on the plot $\bar{\nu }$ = *f*(%_water_, n/n)

The values of *x*_1_ and *x*_2_ were found on the basis of *a*, *b,* and *c* parameters of the polynomial plot according to Eqs. 2 and 3. Since the equation describing the polynomial calibration curve was expressed as

$$\bar{\nu } = ax^{2} + bx + c,$$

the Δ values were calculated for each sample with various water content as follows:

4 $$\Delta_{i} = b^{2} - 4a\cdot(c - \bar{\nu }_{i} )$$

where $\bar{\nu }_{i}$ denoted the position of the absorption band (cm^−1^) of **1** recorded in the water–organic solvent mixture at a given water concentration.

### The effect of the concentration of the sensor

To study the effect of the merocyanine **1** concentration on the results of water determination, the series of three solutions of the dye were prepared in ethanol and DMF (the most polar solvent and the least polar one) in the absence of water as well as in the presence of 2.4% of water. The dye concentration of the particular solutions was 5.88 × 10^−5^ mol L^−1^, 1.18 × 10^−4^ mol L^−1^, and 5.88 × 10^−4^ mol L^−1^, respectively. Then, the electronic absorption spectra were measured using the cells with 1, 0.5, and 0.1 cm path length, respectively, which enabled to obtain the constant value of the product of the path length and the dye concentration. The obtained results revealed that in case of both solvents, with and without water, the increase in dye concentration did not lead to any changes in the position of the recorded absorption band. The maximum of the band was positioned at 17,478 ± 5 cm^−1^, and 17,545 ± 5 cm^−1^ for pure and aqueous ethanol, as well as 14,539 ± 6 cm^−1^ and 15,271 ± 7 cm^−1^ for pure and aqueous DMF. Therefore, it can be concluded that the concentration of the sensor did not influence the position of its absorption band, and as a consequence, the results obtained by the studied method.

### The effect of pH value

The p*K*_a_ value of 5-(5-bromo-6-hydroxynaphthyl-2)-7,7-dimethyl-7H-indolo[1,2-a]quinolinium perchlorate is 7.27 (Sawicka et al. 2010). At pH lower than p*K*a, the dye exists in the solution in the form of the salt which does not exhibit solvatochromic properties. To apply the dye as a water sensor, the pH higher than 7.27 is needed, at which solvatochromic merocyanine is generated in the solution. We obtained merocyanine form of the sensor in situ by the addition of anhydrous K_2_CO_3_ to the sample (5 mg/2 mL). This inorganic salt is poorly soluble in organic solvents, but its solubility can increase after addition of some amounts of water. In case of the samples with the water content below 10%, only a small portion of the salt dissolved and the excess was present in the cell as a solid. Different amounts of water in water–organic solvent binary mixture led to the changes in K_2_CO_3_ concentration and, as a consequence, in pH values of the sample. To check if the changes of pH values influenced the results obtained by the studied water sensor, five aqueous solutions of K_2_CO_3_ were prepared, the concentration of which was 1, 2, 3, 4, and 5 mg/2 mL, respectively. The pH values of these solutions changed from 10.88 to 11.36. Then, the solutions were mixed with the ethanolic solution of the perchlorate form of merocyanine **1** in the ratio 1:1 and 1:3 and UV–Vis spectra were recorded. The experiment revealed that in the whole studied range of pH values, only one absorption band was noticed, which can be attributed to the merocyanine **1**. In case of 50% water–ethanol mixtures, the positions of the bands were 18,931, 18,939, 18,939, 18,932, and 18,932 cm^−1^, whereas in 25% solutions, the bands were positioned at 18,406, 18,399, 18,409, 18,406, and 18,409 cm^−1^, respectively. Therefore, one can admit that the amount of the salt used in the studies enabled to reach the pH value which is enough to generate the solvatochromic merocyanine form of the sensor. Moreover, small changes in the amount of K_2_CO_3_ dissolved in the sample, and, as a consequence, in the pH value, did not influence the position of the absorption band of merocyanine **1**.

### The effect of impurities

The influence of the most common impurities present in the studied solvents on the position of the absorption band of the merocyanine **1** were also studied. For this purpose, UV–Vis spectra were recorded of the dye solution in pure and 96% ethanol with methanol (0–0.3%), isobutyl alcohol (0–0.02%), acetone (0–0.03%), ethyl acetate (0–0.002%), and acetic acid (0–0.006%). The experiments were also performed in case of propane-1-ol and propane-2-ol taking into account methanol (0–0.02%), ethanol (0–0.02%), acetone (0–0.02%), and acetic acid (0–0.2%). The concentration range of each impurities exceeded the values at which these compounds are present normally in the studied organic solvents. The results revealed that the impurities in the studied concentration range did not influenced the position of the absorption band of the water sensor. The changes in the obtained band position did not exceed the instrumental error. The only exception was acetic acid. When its concentration exceeded 0.004%, the intensity of the absorption band corresponding to the solvatochromic merocyanine decreased and a new band appeared, positioned at 24,721 cm^−1^, and attributed to the salt (Sawicka 2015). The spectral changes were caused by the shift of the equilibrium in the solution between the salt and the merocyanine form of the dye (Scheme 1). As a result, the salt without solvatochromic properties started to prevail in the solution and the increase in water content did not lead to any changes in the absorption band position.

### Determination of water content

The validity of the method was verified by determining the water content in various organic solvents. The results were found based on the linear plots in case of ethanol, propan-1-ol, propane-2-ol and DMSO as well as the polynomial curve determined for DMSO and DMF. In all cases, the plots of the absorption band position of **1** versus water content expressed as mole percent were used and the obtained values of mole percent of water were recalculated into mass percent. The results are given in Table 5.

**Table 5:** Results of the test of the applicability of the method for the determination of water content in organic solvents

Solvent	$x_{{{\text{H}}_{2} {\text{O}}}}$ given/%	$x_{{{\text{H}}_{2} {\text{O}}}}$ found/%	Recovery/%
Ethanol	1.25	1.29 ± 0.03	103.10
	4.82	4.88 ± 0.05	101.13
	9.20	9.09 ± 0.06	98.72
Propane-1-ol	1.22	1.25 ± 0.02	101.72
	4.74	4.67 ± 0.05	98.54
	90.5	9.26 ± 0.05	102.32
Propane-2-ol	1.26	1.30 ± 0.01	102.82
	4.87	4.86 ± 0.04	99.83
	9.29	9.29 ± 0.03	100.03
DMSO	0.90	0.90 ± 0.01	99.78
	3.51	3.63 ± 0.01	103.50
	6.78	6.57 ± 0.03	97.04
DMSO^a^	0.90	0.92 ± 0.03	101.76
	3.51	3.52 ± 0.01	100.45
	6.78	6.80 ± 0.04	100.29
DMF^a^	1.04	1.07 ± 0.01	102.88
	4.04	4.05 ± 0.02	100.36
	7.76	7.68 ± 0.02	98.89

^a^The results obtained with the application of polynomial plots

The obtained values proved that the proposed method can be successfully applied to the determination of water content in organic solvents. It concerned both the linear plots and the polynomial ones.

## Conclusions

7H-indolo[1,2a]quinolinium merocyanine proved to be an useful sensor of water in organic solvents. The calibration plots describing the position of the absorption band of the dye as a function of mass or mole percent of water were found at the water content ranging from about 0.05 to 11% (w/w). The method sensitivity depended on the difference in the polarities between an organic solvent and water. Another factor which influenced the method sensitivity was the extent of the preferential solvation of the sensor by water.

Based on the data presented in the manuscript, one can admit that the decrease in the polarity of the solvent leads to the increase in the sensitivity of the water determination. The spectral changes were the most evident in case of the least polar DMSO and DMF, which both are aprotic and do not possess the ability to be a good hydrogen bond donor. On the other hand, these features are responsible for weak interactions of these two organic solvents with water as well as those with the dye molecules. As a result, the preferential solvation of the sensor by water can be noticed and the polynomial calibration curve rather than linear one should be applied.

The validity of the method was tested. It proved that the method can be a useful tool in the determination of water in the studied organic solvents. It is simple, convenient, fast, and suitable for real-time analysis. The applied water sensor enables to obtain very good results, comparable with the ones obtained with some fluorescent probes. Moreover, the measurements can be carried out using UV–Vis spectrophotometer which appears to be a routine equipment in both organic and analytical laboratories. Furthermore, the changes in UV–Vis absorption spectra used in water content analysis usually concern the changes in the value of an absorbance at a given wavenumber which strongly depends on the probe concentration. Therefore, even small inaccuracy in the sample preparation leads to inappropriate results. In case of 7H-indolo[1,2-a]quinolinium merocyanine, the change in the band position was used as a base of calibration curve which is not as much affected by the concentrations of the sensor as it is in case of the absorbance.

## Electronic supplementary material

Below is the link to the electronic supplementary material.

Supplementary material 1 (XLS 55 kb)
